# Gunshot Wounds—Experiences in Civil Life1Read at the 27th annual meeting of the Alumni Association, Buffalo University Medical College, May 2, 1902.

**Published:** 1902-08

**Authors:** William C. Phelps

**Affiliations:** Buffalo, N.Y., 146 Allen Street


### Gunshot Wounds—Experiences in Civil Life.[1]

By WILLIAM C. PHELPS, M. D., Buffalo, N.Y.

WHEN I thought of writing something of my experience in the care of gunshot wounds in civil life, I looked over a rather exhaustive article on this subject and found records of such wounds by the hundreds of thousands, but all occurred in wars, and there was not a word concerning gunshot wounds in civil life. Because of the small proportion of gunshot wounds as compared to others which we are called upon to treat in hospitals and private practice, I find myself in something of the same dilemma as was the boy who was to write a composition on the "Snakes of Ireland" and began with the statement that there were no snakes in Ireland. There are, however, a limited number of wounds from firearms in this locality every year, but they are made by what military surgeons call small arms, rifles and pistols, and an arm that belongs solely to civil life—the shotgun. The pistol and shotgun have caused every such wound that I have had experience with, excepting on a few occasions when the patients have been members of the National Guard or veterans of our late wars. The shotgun is capable of making (and at a close range does make) wounds attended by great mutilation and mortality. I have seen hands entirely shot away and also all the anterior wall of the axilla destroyed. While I have never seen the immediate results of a wound by a fragment of shell in warfare, I think that the shotgun causes similar mutilation.

The pistol wounds have generally been made by a small ball of .22 caliber. I have, however, noticed in recent years that the missile has been of larger size, and by inquiry from officials who carry arms and of others, I have learned that the use of the .22 caliber pistol is more restricted, and that the .38 caliber is the usual weapon now carried. This is to be regretted. The armies of the world have adopted the small bullet of high velocity not because wounds made by it are more fatal, for it is well known that they are not and mutilate the soldier much less than the slow going large bullet of the Springfield rifle type. The object sought to be gained is to put the soldier hit out of action and not to kill him. The same object is accomplished with a small .22 caliber ball in civil life with a minimum of danger of fatal result.

1. Read at the 27th annual meeting of the Alumni Association, Buffalo University Medical College, May 2, 1902.

Police and other officers of the law should not be allowed to carry them, for the only object in their use is to put the person offended in a condition in which he can be captured and not to execute him at the will of the officer without trial as to his guilt or innocence. The manufacture of pistols of large caliber should be prohibited by law.

The majority of pistol wounds we treat are the result of accident or suicide, and in either case it is evident that the less dangerous the wound the better it is for the public at large. In the treatment of gunshot wounds military surgeons claim that surgeons in civil life have a great advantage over them, and should, therefore, obtain better results. The claim is made on the supposition that the surroundings of the average patient are more favorable than those of the soldier on the field of battle. In the very first period of time after the shooting, this may be true in many cases, because of the length of time which may elapse before surgical attention is received, but when that period has passed, I think that the advantage is with the soldier in an army with a medical corps organised and equipped according to present regulations. He is taught the use of aseptic dressing; the ambulance corps arrives with the same knowledge and he is delivered to the operating surgeon with a wound uncontaminated. In civil practice exactly the opposite conditions exist. In the majority of cases we start with an inoculated wound made so by officious but well meaning friends. We always find some dressing applied and its chief constituent is a handkerchief or some equally filthy article of apparel.

During the greater part of my past experience in the practice of accidental surgery no case of gunshot wound has been allowed to get very far away from the locality where it was received without a probe is made, to follow as accurately as possible the track which the missile made for itself through the tissues.

The masters of our profession taught that this is the essential thing to do and that it be done at once. We all followed their instructions and did our best to inoculate the wound, using the same methods that the bacteriologist now uses to inoculate his culture tubes and often with the most deadly pathogenic bacteria, for our probes were never sterilised.

Dr. Oliver Wendell Holmes wrote a beautiful poem in which he likened the medical profession to a light-house tower which for twice a thousand years had withstood all the attacks which its enemies had directed against it. But, if any of them had known of the terrible effects of its teaching concerning the use of the septic probe in gunshot wounds, they would have had a means of attack of the most formidable character; but thanks to our textbooks and journals the knowledge of this error has been spread broadcast through the ranks of our craft and one now sees but little use of the probe, and then only under proper conditions. These conditions are that the patient should be prepared for operation if one is thought necessary, and the probe is to be considered as simply one of the sterilised instruments required.

Of wounds treated in the late civil war 10.77 per cent. were of the head, face and neck; 18.37 of the trunk; 37.70 of the upper extremities and 35.15 of the lower extremities. In civil life I think that the percentage for head, face and neck and upper extremities is much greater than the above given. Wounds of the head are very frequent and they may be of trifling consequence or most serious. Some treated were only scalp wounds which healed by adhesion or if the outer table was injured a granulating wound follows.

When the pistol was of large caliber and fired at a right angle to the contour of the skull more or less penetration occurred. Some of them extended only through the outer table, and if kept aseptic heal kindly. If, however, pus formed, septic meningitis and death were apt to result. When complete perforation took place and the bullet lodged in the brain, death uniformly followed, though in one case some years previously the patient was shot on the left side of the head posterior to and above the ear. The bullet penetrated the brain for about one inch and was removed. The wound healed and remained so about eight years. He entered the General Hospital complaining of pains over the cicatrix with fever, chills and delirium. An exploratory incision was made and a large pus cavity was found. This was cleaned and drained, but coma and death followed in about three days. Neither the operation nor a post-mortem examination determined the case of the abscess after the great length of time since the receipt of the wound.

In another case of penetrating wound with lodgment of bullet in the brain, I was able to locate it and remove it.

A suicide shot himself in the forehead to the left of the median line. He was kept at home for some ten days and until symptoms of severe brain trouble developed, when he entered the General Hospital. The wound was septic. With a Girvin's telephone probe, which Dr. Park kindly loaned, the ball was located one inch from the back of the skull, having gone nearly through the brain in a direct line. A trephine opening was made posteriorly over its location and the brain substance penetrated by forceps when it was grasped and removed. Thorough drainage was established, but the cerebritis continued and ended in death.

I found the Girvin probe of great value in locating the bullet. It was impossible by any other probe to do so, because the processes of the dura mater projected into its tract and gave to the ordinary probe the sensation of a foreign body. As the rule laid down in the books is that bullets or other foreign bodies lodged in the brain substance should be removed when possible, this probe is of great assistance in making certain their location.

Wounds on the neck and face have done well by the usual dressing and no interference by way of operating unless a large vessel nerve was injured. I believe this is the best treatment, and I have been much surprised at the rapidity with which they have closed. When a pistol ball enters a joint there is so little outward appearance of injury that one is tempted to wait and take the chances on the result. I have had occasion to regret following this inclination. A strong, healthy man received a .22 caliber bullet at the lower end of the patella, which penetrated the joint. The wound was so small that the patient considered it a trivial matter. He, however, consented to hospital treatment. Synovitis followed with formation of pus and septicemia. The joint was exsected, but too late to save his life.

I shall in future treat gunshot wounds of the joints in the same manner that wounds of the abdomen should be treated. I shall make exploratory incisions, and having learned the extent of the injury treat it according to general principles, though partial or complete excision with drainage will generally be required. I have come to regard them as exceedingly dangerous, no matter how small the bullet.

As regards bullet wounds of the long bones, I have several patients walking the streets with them imbedded in the bones and they say that they suffer no inconvenience whatever. It is not necessary, therefore, to undertake their removal. Aseptic treatment is all that is required.

Penetrating wounds of the abdomen have been cared for according to the accepted rules of the day—laparatomy and repair of damaged organs, excepting in one instance when the old expectant plan was followed. I read in some medical work on the subject, that bullets could penetrate and traverse the abdominal cavity with little risk of injuring any viscus when it entered at or near the median line, an inch or two above the umbilicus.

I was called to see a case at the Fitch Hospital, the attending staff of the hospital happening to be out of town. The man had received a small pistol ball in the locality described. He had been injured some hours previous to my visit. I found no pain present; pulse, temperature and all functions normal, and he said he was feeling well enough to go home. There was no doubt of penetration of the abdominal wall, this fact having been ascertained by the probe, but because of the location of the wound and his apparently normal condition, I advised waiting until morning and longer if no untoward symptom arose. The advice was followed and the next day he was so well that the Fitch surgeons hesitated to make a laparatomy, and it was not done that day and never was required, for in a few days the patient went home perfectly well.

I do not advocate this method of treating penetrating wounds of the abdomen, for I know of no way of assuring one's self that in a given case the bullet has not wounded intestine or other organ. The case, however, is of interest as it seems to support the assertion that because of the anatomical arrangement of the viscera a penetrating wound above the umbilicus may be devoid of danger.

146 ALLEN STREET.

